# Treatment of lung cancer by acupuncture combined with medicine based on pathophysiological mechanism: A review

**DOI:** 10.1097/MD.0000000000037229

**Published:** 2024-02-09

**Authors:** Chenguang Guan, Hong Chen, Haipeng Chen, Shuhua Li, Yuhan Chen, Jingyu Chen, Yushan Dong, Zhitao Zheng, Kaiwen Wang, Chuqiao Pan

**Affiliations:** a Heilongjiang Academy of Chinese Medicine, Harbin, Heilongjiang Province, China; b Cancer Hospital, Peking Union Medical College, Chinese Academy of Medical Sciences, Beijing, China; c North Hospital of Qiqihar First Hospital, Qiqihar, Heilongjiang Province, China; d Guang'anmen Hospital, China Academy of Chinese Medical Sciences, Beijing, China; e Heilongjiang University of Chinese Medicine, Harbin, Heilongjiang Province, China; f Qiqihar First Hospital South Hospital, Qiqihar, Heilongjiang Province, China.

**Keywords:** Chinese medicine, combination of acupuncture and medicine, lung cancer, SCLC

## Abstract

Lung cancer is one of the most frequently diagnosed cancers in the world. There are an estimated 2.2 million new cases and 1.79 million deaths each year. Over the past 2 decades, our understanding of disease biology, the use of predictive biomarkers, and improvements in therapeutic approaches have made significant progress and transformed the outcomes of many patients. Treatment is determined by the subtype and stage of the cancer; however, the effect of personalized treatment remains unsatisfactory. The use of Chinese medicines has attracted increasing attention worldwide. Chinese medicine treatment of lung cancer has few side effects, which can effectively prolong the survival expectation of patients and improve their quality of life, and has attracted increasing attention. Based on the pathophysiological mechanism of lung cancer reported in modern medical research, this article explores the efficacy and safety of acupuncture combined with medicine in the treatment of lung cancer.

## 1. Introduction

Lung cancer is the leading cause of death worldwide.^[1–3]^ It is estimated that there are 2.2 million new cases and 1.79 million deaths each year.^[4]^ 80% to 90% of lung cancer cases occur in smokers, and smoking is the main risk factor for this disease.^[3]^ The World Health Organization classifies lung cancer into 2 subtypes: non-small cell lung cancer (NSCLC) and small cell lung cancer (SCLC). The former is the cause of approximately 85% of lung cancer cases, while the latter accounts for the remaining 15%.^[5]^ NSLCLC include lung squamous carcinoma (LUSC), lung adenocarcinoma, and large cell carcinoma subtypes.^[5]^

Although the medical community has made important efforts to reduce the mortality rate of lung cancer.^[6–8]^ For example, the use of specific tyrosine kinase inhibitors (TKI) in molecularly defined NSCLC has significantly improved the clinical efficacy in 15% to 20% of patients. Immunotherapy with anti-PD1/PD-L1 antibodies has become a major breakthrough in the treatment of NSCLC.^[9–11]^ However, drug resistance in primary and secondary lung cancer eventually leads to the failure of targeted therapy in all patients.^[12,13]^ Traditional has been shown to have a certain effect on lung cancer. With the emergence of high-quality randomized controlled trials (RCTs), their efficacy and safety have gradually been verified. Based on the pathophysiological mechanism of lung cancer, this study explored the treatment of lung cancer using acupuncture combined with medicine.

### 1.1. Screening for lung cancer

Implementing a screening program to diagnose patients at an early stage is one of the major steps to gradually reduce lung cancer-related deaths and improve survival. Historically, screening studies have used chest radiographs. No improvement in patient outcomes was shown.^[14]^ In the National Lung Screening Trial,^[15]^ approximately 50,000 patients with a history of high-risk smoking were randomly assigned to the screening group to undergo annual low-dose CT or chest radiography for 3 years. The smaller NELSON study^[16]^ randomly assigned men at a high risk of lung cancer to a baseline low-dose CT scan, followed by 4 scans or no intervention over 15 years. Both the National Lung Screening Trial and NELSON studies have shown a significant reduction in lung cancer mortality.

Despite the high rate of false positives and the risk of radiation, available evidence shows that there is no doubt that screening causes a reduction in cancer mortality. This is despite evidence that women are more likely to benefit from screening than are men. However, women were still a minority in the study population. In addition, current US screening recommendations primarily use smoking history to identify high-risk patients.^[17]^

**Figure 1.**
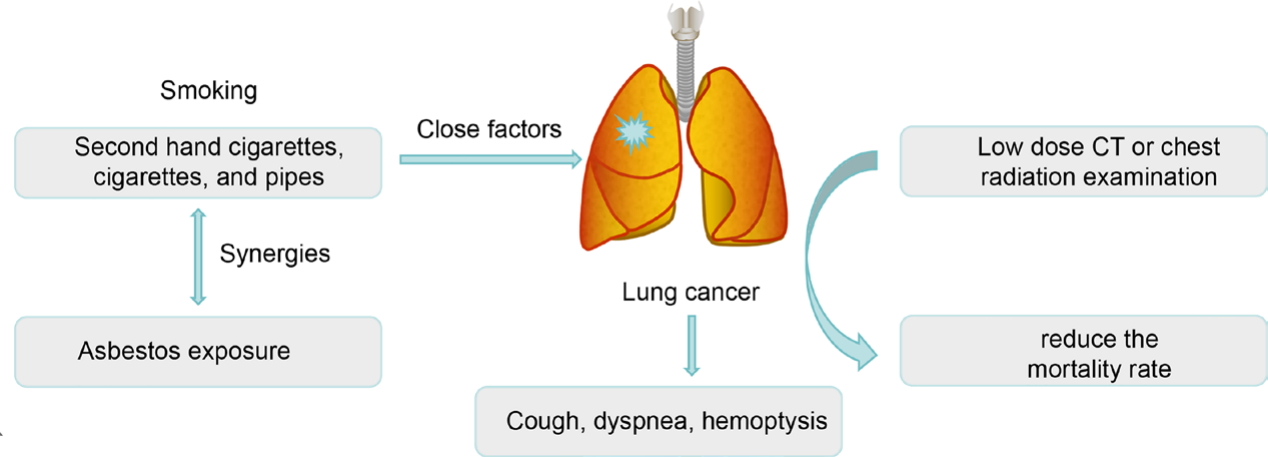
Screening for lung cancer.

This is despite the fact that the improvement in lung cancer survival rates since 2013 is mainly due to new treatments and not screening. However, this was mainly because of the low screening rate.^[4]^ Survival in lung cancer is expected to improve as screening is phased in, especially if ongoing trials help personalize work and optimize screening intervals based on initial scan results.^[18]^ However, the obstacles include the pressure to quit smoking and the cost of screening. Coordinated efforts by healthcare providers and governments are critical for realizing the full potential of screening (see Fig. 1).

### 1.2. Epidemiology of lung cancer

Lung cancer remains the most common cause of cancer death.^[19]^ It is estimated that, in 2018, there were 230,000 new cases in the United States alone. Lung cancer causes more deaths than breast, prostate, and colon cancers.^[20]^ Lung cancer is relatively rare in the first 50 years of life; thereafter, the risk increases with age.^[20]^ Men were more affected than women were. Interestingly, smoking is the risk factor most strongly associated with lung cancer. However, only 15% of smokers develop lung cancer, which indicates a genetic predisposition.^[21]^ Smoking intensity and lifetime duration proportionally increase the risk of impact.^[22]^ Smoking cessation can reduce the risk of lung cancer. For patients who cannot quit smoking completely, even a reduction in the number of cigarettes smoked per day has clear benefits.^[23]^ In general, smoking, including secondhand smoke, cigarettes, and pipes, increases the risk of lung cancer.^[24]^ Due to conflicting results, the association with marijuana is unclear, and its association with e-cigarettes is uncertain. This is partly due to the confounding effects of previous or concurrent cigarette use and lack of long-term data.^[25]^ Asbestos exposure has a synergistic effect with smoking, with a higher incidence of lung malignancies than either risk factor alone.^[25]^ Other risk factors include some form of interstitial lung disease. The presence and family history of COPD were also associated with lung cancer, even after adjusting for tobacco exposure.^[24]^

### 1.3. Clinical manifestations of lung cancer

Cough occurs in approximately half of patients with lung cancer, and new cough symptoms in smokers or former smokers should be of concern for lung cancer.^[26]^ Approximately one-third to one-half of patients with lung cancer experience breathing difficulties. Patients are also at risk of pulmonary embolism, pneumothorax, pleural effusion, and pericardial effusion. Other less common symptoms include chest pain due to local tumor invasion and hoarseness due to recurrent laryngeal nerve involvement. Hemoptysis is a major symptom in approximately a quarter of lung cancer patients and is rarely associated with massive hemoptysis.^[27]^ Other manifestations of intrathoracic spread are superior vena cava syndrome, dysphagia, or arm/shoulder pain. This is due to various structural mass effects. Patients may also develop extrathoracic metastases. These are often nonspecific, and include weight loss, anorexia, and fatigue.^[26]^ Bone metastases are frequently painful, while brain metastases can also be asymptomatic, although neurological sequelae occur depending on size and location. Finally, paraneoplastic syndromes can occur, including syndrome of inappropriate antidiuretic hormone secretion, neurological syndromes such as Lambert-Eaton myasthenia syndrome and cerebellar ataxia, and hypercalcemia due to bone metastases or parathyroid hormone-related protein secretion.^[28]^

### 1.4. Diagnosis and classification of lung cancer

All patients with known or suspected NSCLC should undergo thorough clinical and chest CT examinations.^[29]^ If there are no extrathoracic abnormalities on clinical evaluation and CT scan, PET is recommended to evaluate metastasis.^[7]^ It is important to note that if the primary lung lesion is a ground-glass opacity or a surrounding nodule of ≤3 cm, a PET scan may not be required. This is because these transfers are less likely.^[29]^ These patients can be treated directly as soon as the tissue is diagnosed. Internal iliac node staging is recommended for patients who do not clearly have early or advanced NSCLC. However, invasive staging may not be necessary if the mediastinum is extensively infiltrated.^[29]^ When sampling is required, endobronchial ultrasound-guided bronchoscopic sampling is recommended instead of mediastinoscopy for surgical staging. Direct comparison trials have shown that endobronchial ultrasound-guided thoracic lymph node sampling is as good or better than surgical sampling, although it is less invasive and has fewer complications.^[30]^ However, if bronchoscopy results are negative and lymph node involvement is suspected, surgical confirmation is required.

### 1.5. Epigenetics of lung cancer

The study of epigenetic abnormalities provides key data for understanding lung cancer.^[31]^ Aberrant DNA methylation promotes carcinogenesis by silencing the expression of tumor suppressor genes (TSG) through promoter methylation.^[32,33]^ DNA demethylation is a hallmark of lung cancer and an early event in carcinogenesis.^[34]^ Three DNA methyltransferases (DNMTs) catalyze DNA methylation at the CpG site 5 ^ cytosine. DNMT1 is involved in maintaining established DNA methylation patterns in dividing cells. DNMT3a and DNMT3b are methyltransferases that establish novel methylation patterns by targeting normally unmethylated CpG sites.^[35]^ DNMT1 expression is increased early in lung cancer and is directly involved in the silencing of multiple genes involved in lung cancer pathogenesis, such as RASSF1A and CDKN2a.^[36,37]^ DNMT1 and DNMT3b cooperate to establish aberrant DNA methylation and chromatin complexes to inhibit TSGs.^[38]^ DNMT3b overexpression accelerates carcinogen-induced cell line transformation, is frequently observed in lung cancer, and is associated with poor prognosis.^[39,40]^

The most studied TSG in lung cancer development is CDKN2A, which encodes p16INK4a and p14arf. P16INK4a is a cyclin-dependent kinase inhibitor that plays a key role in cell cycle arrest in the G1/S phase.^[41]^ CDKN2A was the first TSG to be inactivated in lung cancer mainly through aberrant hypermethylation.^[42]^ Methylation of CDKN2A is detected early in precancerous lesions and results in loss of p16INK4a expression.^[43]^ CDKN2A methylation occurs at the earliest antecedent of LUSC, basal cell hyperplasia, which progresses to dysplasia and carcinoma in situ.^[44]^ There is an increased prevalence of loss of heterozygosity involving the 3p locus of FHIT, which is common in NSCLC.^[45]^ FHIT methylation is the main mechanism of FHIT loss, and its expression differs from that of loss of heterozygosity, which can be detected before tumor lesions. It is also associated with squamous epithelium.^[46,47]^

**Figure 2.**
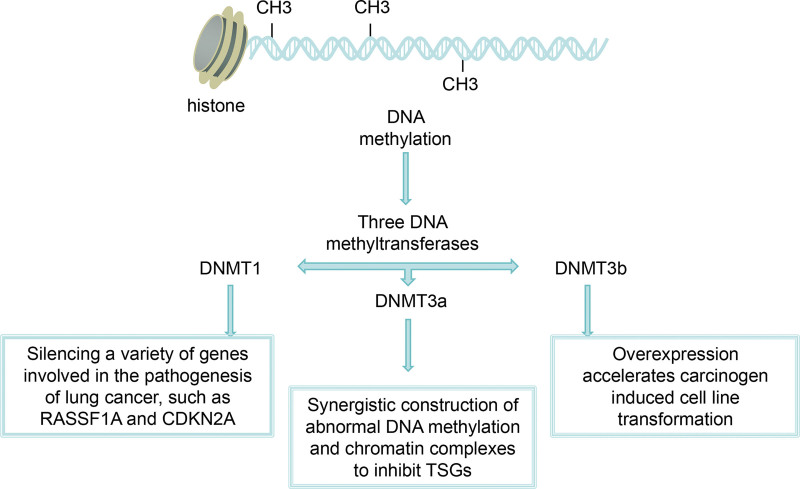
Epigenetics of lung cancer.

Recent studies have shown an increased frequency and levels of gene promoter hypermethylation. Many genes are involved in diverse cellular functions, such as CDKN2A, DAPK (changes in apoptosis), MGMT (DNA repair genes), RARb (retinoic acid signaling), rassf 1A (Ras signaling), and hTERT (immortalization), in the progression from normally-appearing lung to atypical adenomatous hyperplasia and eventual adenocarcinoma^[48,49]^ (see Fig. 2).

### 1.6. Pathophysiological mechanisms of lung cancer

Lung cancer is a heterogeneous disease with a wide range of clinicopathological properties.^[50]^ In the NSCLC classification, adenocarcinoma is the most common subtype of lung cancer,^[51]^ followed by LUSC.^[51]^

The expression of PD-L1 on the surface of tumor cells, detected by immunohistochemistry, is a predictive biomarker to guide the treatment decision for anti-PD-1 or anti-PD-11 antibodies in NSCLC patients. PD-L1 expression is associated with an increased likelihood of response to PD-1 pathway blockade, but response to ICIs is also observed in patients without tumor PD-L1 expression.^[52]^ Moreover, PD-L1 expression is heterogeneous, both within and between tumors.^[53]^ Despite these limitations, tumor PD-L1 expression should be evaluated in all newly diagnosed patients with advanced NSCLC. This can guide the use of ICIs and identify patients who prefer chemoimmunotherapy.

A high TMB predicts a response to ICIs, although there has been no prospective validation.^[54]^ TMB testing is currently not recommended for patients with NSCLC or SCLC. However, according to the US Food and Drug Administration, pembrolizumab, a PD-1 inhibitor, has been approved for use in pretreated patients with high TMB, which deserves special discussion. The results of a preplanned analysis of 10 cohorts of approximately 700 patients showed an improvement in overall response in the group with high TMB compared to the group without high TMB in people treated with pembrolizumab.^[55]^ A higher proportion of patients with high TMB survived 3 years after the first dose of pembrolizumab than patients without high TMB.

The discovery of EGFR precursor cell activating mutations is the first indication that some NSCLCs have oncogenic driver mutations that confer sensitivity to TKI. Oncogene-driven lung cancer follows a common biological framework. Oncogenic drivers are altered, leading to the constitutive activation of kinase signaling pathways that normally require ligand-dependent activation.^[56]^ This appears to be an early clonal event in tumor evolution and is maintained in all subclones that develop during tumor development.^[57]^ It is mutually exclusive of other drivers.^[58,59]^ It also leads to oncogene addiction, in which cancer cells rely on activated signaling pathways to survive. These features form the basis for the direct use of TKI against these carcinogenic factors.

Despite the success of targeted therapies, drug resistance is inevitably emerges.^[60]^ Acquired resistance can be divided into 3 categories: Targeted resistance describes alterations in target genes, including target gene amplification or second-site mutations that interfere with drug binding. Although the target kinase is continuously inhibited, off-target resistance frequently occurs through the reactivation of downstream oncogenic signaling pathways. The third category is phenotypic transformation, in which biopsies performed on patients during disease progression with targeted therapy show transformation from NSCLC to SCLC.^[61,62]^

### 1.7. Cognition of lung cancer in traditional Chinese medicine

In ancient Chinese medicine, lung cancer was called “Xi Ji” or “Lung Ji.” It is recorded in “Plain Questions”^[63]^: “The name of the disease is Xi Ji.” “Sheng Ji Zong Lu” records: “Where the accumulation of gas in the right flank, as big as a cup, lung accumulation also. “When the Qi goes up to the heart, the breath is somewhat harmful, so it is called the breath.” According to its symptoms, it can be concluded that lung cancer belongs to the category of “Xi Ji” and “Xi Ben.” There are also relevant studies in ancient books on the pathogenesis and prognosis of lung cancers. It is recorded in the Treatise on the Causes and Symptoms of Various Diseases^[64]^: “Accumulation is caused by the disharmony between yin and Yang, the weakness of the viscera, the pathogenic wind, and the qi of the viscera.” “Jing Yue Quan Shu” put forward^[65]^: “Where the spleen and kidney deficiency, and weak disorders, many people have the disease of accumulation’’. Li Gao, one of the 4 great masters of the Jin and Yuan Dynasties, believed^[66]^: “Accumulation begins with anger, joy, sorrow, and fear, or with sour, sweet, pungent, and salty taste, or with cold, cold, and hot drinks, or with wind, cold, summer, dampness, dryness, and fire...” If it is prolonged for a long time, it will not disappear, and it will become 5 accumulations. It is recorded in Za Bing yuan Liu Xi Zhu^[67]^: “Pathogenic factors accumulate in the chest, blocking the airway, and qi is blocked, resulting in phlegm and blood, both of which have to compete with the healthy qi...” “When the evil has prevailed, the right will not be able to control it, so it will form a shape and have a block.” From this, we know that the occurrence of this disease is related to 6 exogenous pathogens, deficiency of healthy qi, excessive emotions, improper diet, phlegm, blood stasis, and other factors.

### 1.8. Etiology and pathogenesis of lung cancer

Traditional Chinese medicine believes that the overall nature of lung cancer is caused by deficiencies, mixed deficiencies, and excess. Lung cancer is first caused by the deficiency of vital qi, which is due to the imbalance of yin and yang, and the invasion of the 6 exogenous pathogens by taking advantage of the deficiency, resulting in the stagnation of pathogenic factors in the lung, resulting in the disharmony of lung qi and the failure to disperse and descend. When Qi movement is unfavorable, blood circulation is blocked, and body fluid is out of balance and condenses into phlegm. Phlegm congeals and qi stagnate, and blood stasis blocks channels and collaterals, resulting in lung accumulation over time. “Deficiency,” “phlegm,” “stasis,” and “toxin” are the 4 major pathogenic factors of lung cancer, which run through the whole disease process.

Lung cancer is located in the lung and is closely related to lung, spleen, and kidney dysfunction. Deficiency of the lung and spleen qi is the underlying cause of lung cancer. In the early stages of the disease, phlegm and blood stasis in the lung are more common, and the treatment is mainly to ventilate the lung, regulate qi, resolve phlegm, and remove blood stasis. In the middle stage of the disease, the spleen qi is damaged, and transportation and transformation are abnormal, resulting in the accumulation of phlegm and dampness. Therefore, syndrome differentiation should be based on lung and spleen qi deficiency or spleen deficiency and phlegm dampness, and treatment should be based on supplementing qi and strengthening the spleen, strengthening the earth, and generating metal. With the development of the disease, Qi and Yin are consumed, and the kidney is damaged by deficiency, resulting in deficiency of both Qi and Yin and deficiency of kidney-yang. Therefore, treatment should focus on supplementing Qi and nourishing Yin, warming Yang, and tonifying the kidney.

“Phlegm,” “blood stasis,” and “toxin” are the important pathological characteristics of lung cancer in the aspect of pathogenic excess. Because of the dysfunction of the lung, spleen, and kidney, body fluids fail to distribute and warm, resulting in the accumulation of dampness and phlegm, and the accumulation of phlegm and blood stasis. Therefore, Chinese medicine treatment not only strengthens body resistance, but also need to take into account the eliminates pathogens, including flexible use of phlegm and dampness, promoting blood circulation and removing blood stasis, detoxification and resolving masses, and other treatment methods.

## 2. Mechanism of traditional Chinese medicine in treating lung cancer

Traditional Chinese medicine for SCLC. Huang et al^[68]^ found that solanine extracted from *Solanum nigrum* could inhibit the proliferation of H446 cells and induce apoptosis of tumor cells by upregulating the expression of apoptosis-related proteins such as BAX and CASP3 and downregulating the expression of the antiapoptotic protein BCL2. And the experiment showed that the apoptosis of tumor cells was particularly obvious after H446 cells were treated with 13.6 μmol/L for 24 hours. Guo Huimin et al^[69]^ reported that Paris saponin II combined with camptothecin, a chemotherapeutic drug, acted on H446 cells of SCLC, and found that Paris saponin II could increase the sensitivity of camptothecin to SCLC chemotherapy and promote tumor cell apoptosis by upregulating the expression of ERK, AKT, and p38MAPK and downregulating the expression of Bcl-2 protein. Jiang et al^[70]^ showed that Qianjin Weijing Decoction could promote apoptosis by downregulating the expression of cox-2 protein and upregulating the expression of caspase-3 protein in H446 cells in a dose-dependent manner. Zhiying et al^[71]^ showed that Feiyi Pill inhibited the proliferation of SCLC nude mice and induced tumor cell apoptosis in a dose-dependent manner, and when combined with cyclophosphamide chemotherapy, it could further improve the tumor inhibition rate and play a synergistic role.

Treatment of NSCLC with traditional Chinese medicine. According to modern medical research, traditional Chinese medicine can significantly improve and regulate the immune function of NSCLC patients, especially the regulation of cellular immune functions, such as T cell subsets, natural killer cells, interleukin-4, and interleukin-13. Interleukin-13, transforming growth factor-β, interferon-γ, and tumor-associated macrophages. To achieve the purpose of resist tumor recurrence and metastasis, prolong survival time, alleviate adverse reactions to chemotherapy, and improve quality of life.^[72]^ Xu Rongzhong et al^[73]^ found in the study on the influence of different therapeutic principles of strengthening body resistance and eliminating pathogenic factors on the immune function of NSCLC patients that the heat-clearing and detoxifying prescription group and the Qi-tonifying and Yin-nourishing detoxifying prescription group could significantly reduce the expression of regulatory T cells and myeloid-derived suppressor cells in peripheral blood of lung cancer patients, thereby regulating the immune function of lung cancer patients. Lee et al^[74]^ found that ophiopogonin D, the main active ingredient in *Ophiopogon japonicus*, can inhibit NF-κB by inducing IL-6, inhibit p65 nuclear translocation and phosphorylation, inhibit the activation of downstream oncoproteins regulated by AP-1, and reduce the expression levels of COX-2, Bcl-x2, IAP-2, and MMP-9. The combination of ophiopogonin D and paclitaxel enhanced the effect of paclitaxel on apoptosis of lung cancer cells A549. Wang Congcong et al^[75]^ found that curcumin could reduce the number of vascular mimicry formations and significantly reduce the expression of β-catenin gene mRNA and VE-cadherin protein in an experiment to observe the effect of curcumin on the formation of vascular mimicry and the expression of genes related to the Wnt/β-catenin signaling pathway in lung cancer cell A549. Curcumin extracted from *Curcuma longa, Curcuma zedoary*, and *Curcuma aromatica* may inhibit the formation of vascular mimicry in lung cancer cells by inhibiting the Wnt/β-catenin signaling pathway.

### 2.1. Treatment of lung cancer with traditional Chinese medicine

**Figure 3.**
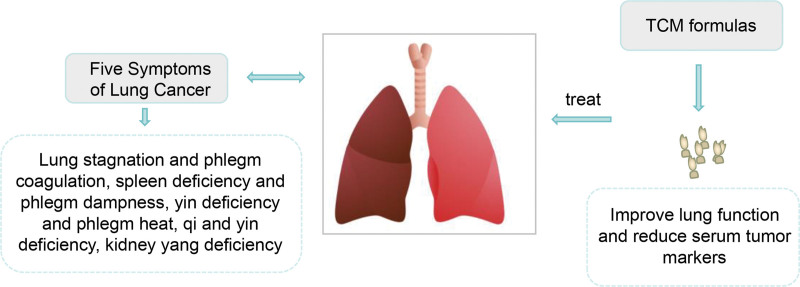
Treatment of lung cancer with traditional Chinese medicine.

According to the Expert Consensus on Diagnosis and Treatment of Lung Cancer with Integrated Traditional Chinese and Western Medicine,^[76]^ lung cancer is currently divided into 5 syndromes in the field of traditional Chinese medicine: lung stagnation and phlegm coagulation, spleen deficiency and phlegm dampness, yin deficiency and phlegm heat, deficiency of both qi and yin, and deficiency of kidney yang (see Fig. 3).

Lixia et al designed a RCT to observe the clinical efficacy of Shenqi Bufei Decoction in patients with lung qi deficiency after thoracoscopic surgery for non-small cell lung cancer.^[77]^ Ninety-four patients were randomly divided into control and observation groups, with 47 patients in each group. The control group received conventional treatment, and the observation group was given Shenqi Bufei Decoction on the basis of the control group, with a course of 3 weeks. The incidence of pulmonary complications, Traditional Chinese medicine (TCM) syndrome score, pulmonary function index (FVC, FEV1, and FEV1/FVC), and SGRQ score were determined. The Shenqi Bufei Decoction can improve lung function, reduce the incidence of postoperative complications, and improve the quality of life of patients with lung qi deficiency after thoracoscopic surgery for non-small cell lung cancer, which has great clinical value.

Xiaohua et al designed an RCT to observe the effect of a modified Shengxian Decoction (Radix Astragali, Rhizoma Anemarrhenae, Radix Bupleuri, Radix Platycodonis, and Rhizoma Cimicifugae) combined with anlotinib on the survival and prognosis of elderly patients with advanced lung squamous cell carcinoma.^[78]^ A total of 148 elderly patients with advanced cell carcinoma were randomly divided into ascending and subsiding groups and a control group n (= 74). The control group was administered anlotinib. The Shengxian group was treated with a modified Shengxian decoction on the basis of the control group. Both groups were treated continuously until the disease progressed or until the side effects of the drugs could not be tolerated. The Short-term clinical efficacy and serum tumor markers (carcinoembryonic antigen [CEA] and cytokeratin 19 fragment antigen 21-1) before and after the 2 courses of treatment were compared between the 2 groups. CY21-1)], Prognostic quality of life (Eastern Cooperative Oncology Group [Eastern Cooperative Oncology Group, ECOG] score, quality of life [quality of life, QOL] score), and adverse reactions during treatment. Survival curves (progression-free survival [PFS] and overall survival) of the 2 groups were plotted. Researchers have found that the application of modified Shengxiantang combined with anlotinib in the treatment of elderly patients with advanced lung squamous cell carcinoma can improve the disease control rate, reduce serum tumor markers, improve the prognosis, prolong the survival cycle, and do not increase the incidence of adverse reactions, which has certain clinical application value.

Gao et al designed RCT to explore the effect of Huoxue Huashi Decoction (Rhizoma Imperatae, Rhizoma Atractylodis Macrocephalae, Radix Paeoniae Rubra, Semen Phaseoli, Radix et Rhizoma Rhei, Radix Codonopsis, Poria, Flos Carthami, Herba Leonuri, Herba Artemisiae Scopariae, Stigma Maydis, Herba Lycopi, and Fructus Gardeniae) targeting TP53 on M2 macrophage polarization and NSCLC cell invasion.^[79]^ TP53, the target of Huoxue Huashi Decoction in the treatment of NSCLC, was screened by network pharmacology analysis. The expression levels of TP53 in NSCLC cells were detected using qRT-PCR and western blotting. THP-1 cells were induced into M0 macrophages, which were treated with Huoxue Huashi Decoction and TP53. The expression of CD86 and CD206 was analyzed using flow cytometry, and the concentrations of IL-1β and CCL18 were detected using ELISA. NSCLC cells were treated with Huoxue Huashi Decoction, TP53 overexpression vector, and macrophage culture medium, and the invasive ability of NSCLC cells was detected by Transwell assay. Huoxue Huashi Decoction inhibits the polarization of M2 macrophages by inhibiting TP53, thus inhibiting the invasion of NSCLC cells.

Liao et al designed an RCT to observe the clinical efficacy of high-dose Jiawei Sijunzi Decoction (Radix Codonopsis, Rhizoma Atractylodis Macrocephalae, Poria, Radix Glycyrrhizae Preparata) in the treatment of elderly patients with advanced non-small cell lung cancer (NSCLC).^[80]^ Ninety elderly patients with advanced NSCLC were randomly divided into control and experimental groups, with 45 patients in each group. The patients in the control group received symptomatic and supportive treatments. On the basis of the control group, the experimental group was given sequential treatment with high-dose Jiawei Sijunzi Decoction, and both groups were treated for 35 days. TCM syndrome scores, Karnofsky functional status scores, and the Chinese version of the Functional Assessment System for Cancer Treatment V4. The 0 scores and immune function indices were compared between the 2 groups before and after treatment, and the incidence of adverse reactions was recorded. Researchers have found that high-dose modified Sijunzi decoction sequential treatment in elderly patients with advanced NSCLC can alleviate the clinical symptoms of patients, improve their quality of life, enhance immune function, and have good safety.

### 2.2. Acupuncture for lung cancer

Guohua et al designed an RCT to explore the effect of timing acupuncture therapy (Geshu, Pishu, Weishu, Shenshu, Zusanli, Sanyinjiao and Dazhui) under the guidance of the theory of “midnight-noon ebb-flow” on the prevention of bone marrow suppression after chemotherapy for lung cancer.^[81]^ A total of 142 patients with NSCLC who received chemotherapy for the first time were randomly divided into a control group (n = 47), acupuncture group (n = 48), and timing group (n = 47) according to the random number table and allocation scheme generated by the computer in advance. The control group received routine nursing care for lung cancer chemotherapy. Based on the measures in the control group, the Geshu, Pishu, and Weishu acupoints were used in the press needling group. In the control group, patients in the timing group were treated with midnight–noon ebb-flow and acupuncture from the first day to the second day. Changes in routine blood indexes and comfort scores reflecting bone marrow suppression were detected and analyzed before chemotherapy and on the 3rd, 7th, 14th, and 21st days of chemotherapy in the 3 groups. Researchers found that midnight-noon ebb-flow acupuncture therapy can effectively prevent bone marrow suppression after chemotherapy in NSCLC patients, improve some routine blood indicators such as white blood cell count, neutrophil count, and platelet count, and enhance the comfort of patients.

Guojiu et al designed an RCT to observe the clinical efficacy of Bo abdominal acupuncture (Zhongwan, Xiawan, Qihai, Guanyuan) in the treatment of patients with advanced non-small cell lung cancer with deficiency syndrome and its impact on immune function.^[82]^ Sixty patients with advanced non-small cell lung cancer with deficiency syndrome were randomly divided into an observation group and a control group, with 30 patients in each group. Patients in the control group were treated with conventional drugs. Based on the treatment of the control group, the observation group was treated with Bo abdominal acupuncture. Once a day, 7 days as a course of treatment, the 2 groups were treated for 2 courses. After treatment and follow-up, PFS, median survival time, and 1-year survival rates were observed. The absolute counts of CD3+ T cells and CD4+ T cells and the ratio of CD4+ T cells to CD8+ T cells (CD4+/CD8+ ratio) were compared between the 2 groups one month before and after treatment. Researchers have found that Bo abdominal acupuncture can improve the survival time and quality of life of patients with advanced non-small cell lung cancer of deficiency syndrome through tonifying methods, such as guiding qi to return to the primordial qi, and can improve the immune function of patients with good clinical efficacy.

He Peishan et al designed RCT to study the clinical efficacy of Laoshi Needle (Zhongwan, bilateral Zhangmen, Qihai, bilateral Tianshu, bilateral Neiguan and bilateral Zusanli) in alleviating fatigue related to argon-helium cryosurgery in elderly patients with advanced NSCLC.^[83]^ Sixty-three elderly patients with advanced NSCLC who underwent argon-helium cryoablation and developed fatigue symptoms were included in this study. The patients were divided into an acupuncture group (n = 32) and a control group (n = 31). The fatigue degree score, fatigue symptom, and quality of life scores (Karnofsky functional status) were compared between the 2 groups. Researchers found that Laoshi Needle can effectively alleviate fatigue related to argon-helium knife cryotherapy in elderly patients with advanced NSCLC, alleviate the symptoms of fatigue, shortness of breath, and lack of appetite, and reduce the impact of argon-helium knife cryotherapy on quality of life, which has positive clinical significance.

### 2.3. Treatment of lung cancer by acupuncture combined with traditional Chinese medicine

**Figure 4.**
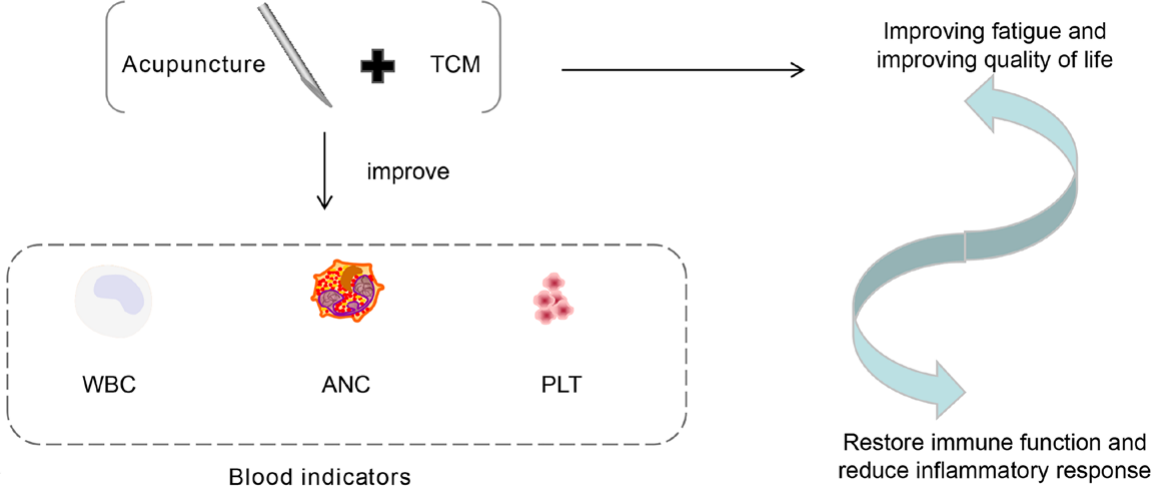
Treatment of lung cancer by acupuncture combined with traditional Chinese medicine.

Qin et al designed an RCT to observe the clinical efficacy of acupuncture (Baihui, bilateral Neiguan, Qihai, and bilateral Zusanli) combined with Hewei Yangxue Prescription (Radix Codonopsis, Rhizoma Atractylodis Macrocephalae, Poria, Rhizoma Pinelliae, Pericarpium Citri Reticulatae, Fructus Aurantii Immaturus, Fructus Amomi, and Endothelium Corneum Gigeriae Galli) in the treatment of cancer-related fatigue in lung cancer with qi and blood deficiencies.^[84]^ Fifty-nine cases of lung cancer patients with cancer-related fatigue were randomly divided into treatment (29 patients) and control (30 patients) groups. The treatment group was treated with acupuncture plus the Hewei Yangxue Decoction. The control group was treated with sham acupuncture plus the Hewei Yangxue Decoction. The Piper Fatigue Scale (revised Piper Fatigue Scale) was used before and after treatment. PFS-R and the European Organization for Research and Treatment of Cancer core quality of life. The changes in questionnaire, European Organization for Research and Treatment of Cancer [QLQ-C30], and clinical efficacy were compared between the 2 groups. Conclusion: Acupuncture combined with Hewei Yangxue Decoction can improve fatigue and quality of life in patients with lung cancer (see Fig. 4).

Liu Zhijuan et al designed an RCT to observe the effects of wrist-ankle acupuncture (upper area 1, located between the flexor carpi ulnaris tendon and the ulnar margin of the little finger; upper area 2, the Neiguan point of the pericardium meridian, located in the center of the palmar side of the wrist; lower area 1, located at the medial edge of the Achilles tendon) combined with Erchen Decoction (Rhizoma Pinelliae Preparata, Poria, Exocarpium Citri Reticulatae, Fructus Mume, Rhizoma Zingiberis Recens, and Radix Glycyrrhizae Preparata) on the immune function and inflammatory factor levels in patients after radical resection of lung cancer.^[85]^ Eighty patients with lung cancer after radical resection were randomly divided into control and observation groups, with 40 patients in each group. Patients in the control group received routine treatment, including oral administration of compound methoxyphenamine capsules and comprehensive breathing training. The observation group was treated with wrist-ankle acupuncture combined with Erchen Decoction compared to the control group. After treatment, the scores of TCM syndromes of cough and wheezing, pain and behavioral status, immune factors, inflammatory factors, pulmonary function, postoperative rehabilitation time, exercise endurance, quality of life scores, and inflammatory factor levels were compared between the 2 groups. Conclusion Wrist-ankle acupuncture combined with Erchen decoction can significantly improve the symptoms and behavioral status of patients with lung cancer after radical surgery, restore immune function, reduce inflammatory reactions, improve lung function, accelerate the rehabilitation process, enhance exercise endurance, and improve the quality of life, with high safety.

Yanhua et al designed an RCT. To observe the curative effect of Runfei Sanjie Decoction (Radix Ophiopogonis, Bulbus Lilii, Ginseng Tablet, Radix Rehmanniae, Herba Solani Lyrati, Pumex, Pericarpium Trichosanthis, Radix Scrophulariae, Rhizoma Pinelliae Preparata, Carapax Trionycis, Concha Ostreae, Squama Manis powder and Radix Glycyrrhizae Preparata) combined with abdominal acupuncture (Zhongwan, Xiawan, Qihai, Guanyuan, bilateral Tianshu, bilateral Daheng, bilateral Huaroumen). The effect of gastrointestinal reactions.^[86]^ Seventy-four patients with advanced cancer were randomly divided into study and control groups, with 37 patients in each group. The control group was treated with the GP scheme and the study group was treated with Runfei Sanjie Decoction combined with abdominal acupuncture. Both groups were treated for 3 consecutive cycles, with 21 days as a cycle. The therapeutic effects, TCM symptom scores before and after treatment, and levels of tumor markers CEA, CA153, and CA199 were compared between the 2 groups, and the effects of the 2 groups on the gastrointestinal tract were compared. Researchers have found that Runfei Sanjie Decoction combined with abdominal acupuncture has a definite effect on advanced lung cancer, which can reduce the expression of tumor markers, improve the symptom score of traditional Chinese medicine, and reduce the gastrointestinal reactions caused by chemotherapy, and is worthy of clinical application.

## 3. Discussion

In addition to the traditional compound and acupuncture treatment of lung cancer, research on the treatment of lung cancer with single traditional Chinese medicine and extracts of traditional Chinese medicine has also made progress.^[87]^ Hong et al explored drugs for early and precise prevention and treatment of recurrence and metastasis of lung cancer and explored the efficacy of Qinglongyi Hospital preparations, which have been approved in China and are currently conducting RCT studies.

TCM has obvious characteristics and advantages in the treatment of lung cancer, and has made remarkable achievements in clinical and experimental research; however, it is an authoritative and systematic system of TCM syndrome differentiation and treatment of lung cancer. In clinical research, there is no real rule for diagnosis and treatment that reflects the advantages of TCM syndrome differentiation and treatment. All patients were blindly included in the study with fixed prescriptions or single drugs, and most of them received chemotherapy combined with traditional Chinese medicine. However, there are few clinical studies based on traditional Chinese medicine or the combination of acupuncture and medicine, and there are some problems such as inconsistent criteria for efficacy, small sample size of clinical observation and no control group, and blind design is even less mentioned. In terms of experimental research, there are many ways and targets of action of traditional Chinese medicine, and the pharmacological mechanism of monomer components is complex; therefore, the mechanism of action of traditional Chinese medicine on lung cancer has not been fully clarified. At the same time, experimental research and clinical research are out of touch, lack communication, some excellent results have not been well transformed, and few of them can really guide clinical application.

The development of molecular biology, systems biology, network pharmacology, and artificial intelligence has provided ideas and technologies for the treatment of lung cancer using Chinese medicine.^[88]^ Future research should focus on the mechanism of traditional Chinese medicine in the treatment of NSCLC, carry out more extensive clinical research of traditional Chinese medicine, find evidence-based basis for the treatment of NSCLC with traditional Chinese medicine, and formulate a unified path of diagnosis and treatment of traditional Chinese medicine, so as to improve the clinical efficacy of traditional Chinese medicine in the prevention and treatment of NSCLC.

The ever-changing research status of lung cancer poses a challenge to the treatment of integrated traditional Chinese and Western medicine under the new situation. For example, the TCM pathogenesis of lung cancer involves many factors such as qi stagnation, blood stasis, phlegm coagulation, and toxin accumulation, which affect the functions of the viscera. This is consistent with the concept of modern medicine that cancer is a systemic disease, but the correlation and regularity between TCM pathogenesis and modern medicine require further scientific interpretation. Another example is that TCM syndrome differentiation of lung cancer is insufficient for objectification, quantification, and standardization. What is the relationship between the syndrome differentiation of lung cancer and the imaging, pathology, and staging of modern medicine? We need to draw lessons from modern technology and methods and carry out a lot of research work from clinical, diagnostic, and other perspectives. At the same time, the diagnosis and treatment norms of integrated traditional Chinese and Western medicine for lung cancer require more evidence-based medical evidence. With the integration of various concepts of Chinese and Western medicine and the continuous development of various means and treatment methods of integrated Chinese and Western medicine, it has become the common goal of traditional Chinese medicine and Western medicine in the treatment of lung cancer to improve survival while considering safety and quality of life. Traditional Chinese medicine needs to give full play to the advantages of individualized treatment and effective low toxicity, pay attention to the accumulation of evidence of evidence-based medicine, combine with disease differentiation and treatment, and constantly complement the advantages of modern medicine to form a more comprehensive and perfect diagnosis and treatment program of integrated traditional Chinese and Western medicine.

## Author contributions

**Conceptualization:** Chenguang Guan.

**Data curation:** Chenguang Guan, Hong Chen, Haipeng Chen, Shuhua Li, Yuhan Chen, Jingyu Chen, Yushan Dong, Zhitao Zheng, Kaiwen Wang, Chuqiao Pan.

**Formal analysis:** Chenguang Guan.

**Funding acquisition:** Chenguang Guan.

**Investigation:** Chenguang Guan.

**Methodology:** Chenguang Guan.

**Project administration:** Chenguang Guan.

**Resources:** Chenguang Guan.

**Software:** Chenguang Guan.

**Supervision:** Chenguang Guan.

**Validation:** Chenguang Guan.

**Visualization:** Chenguang Guan.
